# Preparation and Self-Cleaning Performance of High-Strength Double-Layer PVDF-PVC-Nano-Graphite/PVDF-PVC Super-Hydrophobic Composite Membrane

**DOI:** 10.3390/molecules28237821

**Published:** 2023-11-28

**Authors:** Dashuai Zhang, Shishu Sun, Yan Zhang, Xiaopeng Zhang, Chen Li, Tianyi Sun, Linhua Zhu, Zaifeng Shi

**Affiliations:** 1 Key Laboratory of Water Pollution Treatment & Resource Reuse, College of Chemistry and Chemical Engineering, Hainan Normal University, Haikou 571158, China; zdw198562@163.com (D.Z.); sss_1996@163.com (S.S.); zy936457505@163.com (Y.Z.); zxp_inorganic@126.com (X.Z.); hy0211100@hainmc.edu.cn (C.L.); tianyi870328@163.com (T.S.); zhulinhua1981@163.com (L.Z.); 2 School of Chemistry and Chemical Engineering, Nanjing University, Nanjing 210023, China; 3 Engineering Research Center of Biomembrane Water Purification and Utilization Technology of Ministry of Education, Anhui University of Technology, Ma’anshan 243032, China

**Keywords:** PVDF-PVC composite membrane, nano-graphite, super-hydrophobic, double-layer composite membrane, self-cleaning properties

## Abstract

The double-layer PVDF-PVC (D-PP/PP) super-hydrophobic composite membrane was prepared by the coating immersion phase separation method to enhance the mechanical properties of the composite membrane. The D-PP/PP super-hydrophobic membrane was prepared using the casting solution concentration of 12 wt% PVDF-PVC composite membrane as basement and 4% casting of PVDF-PVC coating. The contact angle of the D-PP/PP membrane was 150.4 ± 0.3°, and the scanning electron microscope showed that the surface of the D-PP/PP membrane was covered by a cross-linked micro–nano microsphere. The mechanical properties showed that the maximum tensile force of the D-PP/PP composite membrane was 2.34 N, which was 19.4% higher than that of PVDF-PVC (1.96 N). Nano-graphite was added to the coating layer in the experiment. The prepared double-layer PVDF-PVC-nano-graphite/PVDF-PVC (D-PPG/PP) composite membrane reached 153.7 ± 0.5°, the contact angle increasing by 3.3°. The SEM comparison showed that the D-PPG/PP composite membrane had a more obvious micro–nano level microsphere layer. The mechanical properties are also superior. By preparing the D-PP/PP membrane, the mechanical properties of the membrane were improved, and the super-hydrophobic property of the coating was also obtained. At the same time, it was found that adding nano-graphite to the coating layer can better improve the hydrophobic, mechanical, and self-cleaning properties of the D-PP/PP composite membrane.

## 1. Introduction 

Polyvinylidene fluoride (PVDF) and polyvinyl chloride (PVC) are widely used in membrane preparation because of their excellent chemical resistance, good thermal stability, and good mechanical properties [1,2,3]. PVDF is soluble in N-N-dimethylformamide (DMF), which is miscible with water. The pore structure and hydrophobicity of PVDF membrane prepared by the simple and practical NIPS method can be well adjusted [4,5]. PVC is one of the most industrialized materials at present, with the advantages of low price, excellent performance, long service life, etc. [6,7,8]. In order to reduce the production cost of PVDF membrane and improve the performance of the membrane, we try to add PVC at a low price and excellent performance to PVDF (the price of PVC is about 1/8 of PVDF). It was found that the hydrophobicity of PVDF-PVC composite membrane can be improved to a certain extent by blending PVDF and PVC, and the production cost of the membrane can be reduced [9,10]. The experiment showed that the hydrophobicity of the composite membrane could be improved by changing the concentration of the casting solution of the PVDF-PVC composite membrane. The contact angle of the composite membrane prepared with 4 wt% PVDF-PVC casting solution can reach more than 150°, but the mechanical properties of the 4 wt% PVDF-PVC composite membrane (M-4) were poor. The 12 wt% PVDF-PVC composite membrane (M-12) prepared with 12% PVDF-PVC casting solution has good mechanical properties [11]. In order to obtain a super-hydrophobic composite membrane with high mechanical properties, a low-concentration PVDF-PVC coating layer was designed on the surface of PVDF-PVC composite membrane with strong mechanical properties to prepare a D-PP/PP super-hydrophobic composite membrane with high mechanical properties.

Super-hydrophobic modification of membrane surface coating is to use low surface energy solution or materials that can form a rough surface to coat on the surface of the base membrane, and finally form a rough and low surface energy composite layer on the surface of the membrane, so as to improve the hydrophobicity of the membrane and even reach super-hydrophobicity [12,13,14]. When super-hydrophobic modification is applied to the surface of the PVDF membrane, the operation is simple, the effect is significant, and the scope of application is wide. Large-scale production can be achieved [15,16,17,18], and the effect on flux and retention is small, which increases the operation cycle of the modified membrane [19,20,21,22,23].

The effects of PVDF solid content in the coating solution and coating conditions on the hydrophobicity of the composite membrane were investigated by Wang et al. [24]. When the PVDF content in the coating solution is 1.88 wt%, the maximum contact angle of the membrane is 136°. The contact angle of the composite membrane increases first and then decreases with the immersion time. When the immersion time is 40 s, the contact angle reaches 133°. The contact angle of the composite membrane decreases with the increase in DMAc content in the coagulation bath and increases with the increase in the coagulation bath temperature. When the coagulation bath temperature is 65 °C, the contact angle of the membrane surface increases to 153°. Wang et al. [16] prepared a super-hydrophobic coating with a papillary structure on the surface of the polyvinylidene fluoride (PVDF) membrane. The surface morphology and water contact angle (WCA) vary with the content of PVDF and polyethylene glycol (PG) in the coating solution, immersion temperature, and time. The maximum water absorption coefficient of the super-hydrophobic surface is 156.8° after being modified in the coating solution of 45 °C, 2 wt% PVDF, and 30 wt% PG for 35 s. Lin et al. [25] prepared the membrane casting solution with a certain proportion of PVDF/DMAC/low molecular polyethylene glycol (PG), prepared the membrane, and then used the polymer solution as the coating solution to coat the membrane. The study found that with the decrease in the concentration of PVDF in the coating solution, the coating solution gradually changed from continuous phase to discontinuous phase, and after coating, the membrane surface formed granular bulges, and the membrane contact angle increased to 153.2°. The addition of PG causes a large number of tiny aggregates to form in the coating solution, resulting in an increase in the contact angle of the composite membrane surface, forming a rough membrane surface, and improving the contact angle of the composite membrane.

The surface modification of PVDF polymer by phase conversion method can improve the hydrophobicity of PVDF membrane [26,27,28]. When preparing a PVDF super-hydrophobic composite membrane with PVDF solution as a coating solution, due to the unique adhesion of PVDF, the solidity of the surface coating and the base membrane is increased [16]. PVDF membrane manufacturing process is simple and easy to control, the surface energy is low, and the thermal conductivity is small, but the price of PVDF membrane raw materials is relatively high, which increases the operating cost in practical applications. In order to reduce the production cost of PVDF membrane and improve the hydrophobic property of the membrane, we can try to add cheap and excellent PVC to PVDF, use PVDF-PVC blend as the coating layer, and use polymer solution as the coating liquid to prepare D-PP/PP super-hydrophobic composite membrane.

In this study, PVDF-PVC composite membrane was prepared by the non-solvent induced phase separation (NIPS) method, and the D-PP/PP super-hydrophobic composite membrane with high mechanical properties was prepared by the coating low concentration PVDF-PVC coating on the surface of PVDF-PVC composite membrane with strong mechanical properties. The membrane material of the same coating solution and the base membrane is PVDF-PVC, the adhesion of the PVDF-PVC body increases the firmness of the surface coating and the base membrane, increases the stability of the membrane and extends the stable operation time of the membrane. Using the same coating solution and membrane material as the substrate, the adhesion of the PVDF-PVC body increases the adhesion between the surface coating and the substrate, increases the stability of the double-layer membrane, and prolongs the stable operation time of the double-layer membrane. The surface roughness of the PVDF-PVC composite membrane was increased by the phase conversion method and by changing the component content of the coating solution. At the same time, it was found that adding nano-graphite to the coating layer can better improve the surface roughness and mechanical properties of the D-PP/PP composite membrane. In addition, the self-cleaning experiment of the D-PP/PP super-hydrophobic composite membrane shows that the composite membrane has a lasting self-cleaning performance. It has potential application prospects in the fields of antifouling and self-cleaning equipment, membrane distillation, and seawater desalination.

## 2. Results and Discussion

### 2.1. Experimental Results of Solvent Concentration Selection

The dissolution of 4 wt% PVDF-PVC (85/15) coating is shown in the experimental results in Table 1. It can be seen that in order to dissolve 4 wt% PVDF-PVC (85/15) powder, the proportion of polyethylene glycol should not be greater than 35%. The dissolution test results of the base membrane are shown in Table 2. In order to prevent the dissolution of polyethylene glycol in the base membrane, the proportion is at least greater than 20%. Comparing the dissolution of the coating layer in Table 1 with the dissolution of the base membrane in Table 2, PG-30 and PG-35 can be considered as the coating dissolution reagent and the base membrane expansion reagent.

Through the dissolution test of the coating material and M-12 base membrane (Table 1 and Table 2), the temperature and solvent (PG/DMF ratio) suitable for the dissolution of the coating and the expansion of the base membrane can be selected. The coating material can be dissolved without dissolving the base membrane at 50 °C, PG/DMF is 30/70, and the coating material can be dissolved without dissolving the base membrane at 60 °C, PG/DMF is 35/65. During the preparation of the double-layer membrane, in order to prevent the deformation of the base membrane at the moment of meeting the solvent and the local shrinkage deformation, the casting agent is generally selected for pre-expansion before the base membrane is scraped. The price ratio of industrial DMF is about one-third of that of PG, and the temperature of 50 °C is more energy-saving than that of 60 °C. From an economic point of view, the conditions for developing the base membrane are 50 °C and PG/DMF ratio of 30/70.

In order to reduce the amount of PG used in dissolving the coating material, the solvent ratio of PG/DMF for dissolving the coating material is 20/80, 25/75, and 30/70 (PG-20, PG-25, and PG-30), respectively, so as to reduce the amount of PG. In order to expand the preparation temperature conditions of the double-layer composite membrane, the casting solution was selected to prepare the double-layer membrane at 60 °C, 50 °C, 40 °C, and 30 °C by controlling the temperature of the casting solution when scraping the membrane.

### 2.2. Base Membrane Expansion Test

In order to prevent the deformation of the base membrane in the solvent, and at the same time, it is hoped that the polymer PVDF and PVC on the surface of the base membrane will have a certain extension in the developing agent, so as to increase the firmness between the two layers, the base membrane expansion experiment is designed. It can be seen from Table 1 that PG-30 and PG-35 can be solvent for the base membrane expansion. In order to reduce the amount of PG used in the base membrane expansion, PG-30 is selected for the solvent experiment of the base membrane expansion. Compared with other temperature solvents, PG-30 has a poor expansion effect on the base membrane at 30 °C. If PG-30 has a certain dissolution and expansion effect on the base membrane at room temperature, the dissolution and expansion effect of PG-30 on the base membrane will be more obvious at 40 °C, 50 °C, and 60 °C. After the expansion of PG-30, measure the surface morphology and contact angle of the base membrane.

**Figure 1:**
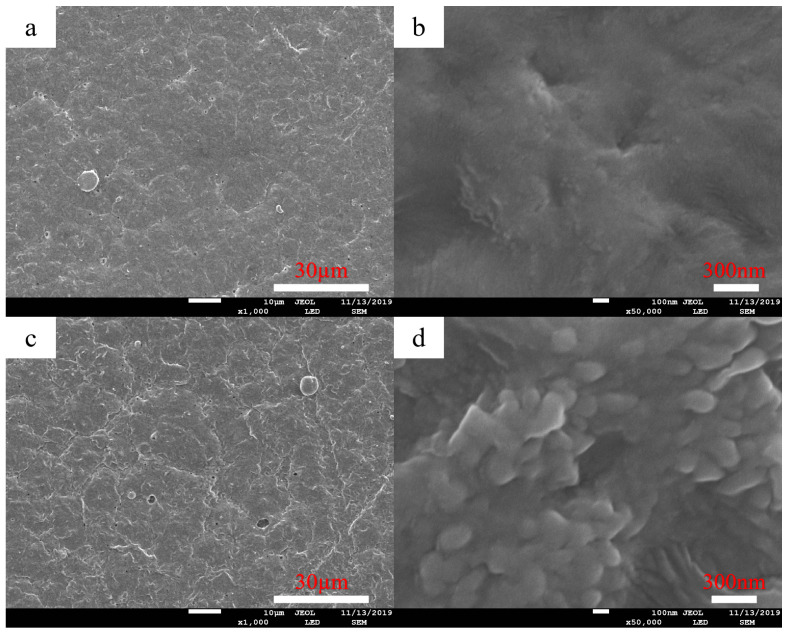
The SEM images of the surface topography of the PVDF-PVC basement membrane (**a**,**b**) and the PVDF-PVC basement membrane after PG-30 unfolding (**c**,**d**). Left, magnification: 5000. Right, magnification: 40,000.

**Figure 2:**
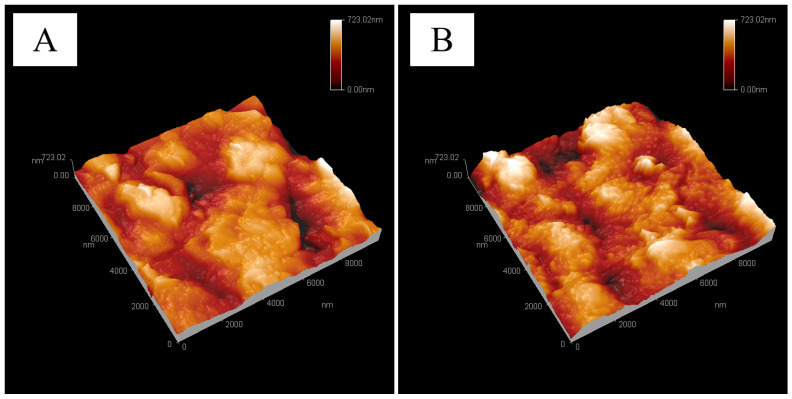
AFM 3D images of the surface topography of the original PVDF/PVC (**A**) and PVDF/PVC basement membrane after PG-30 unfolding (**B**), respectively.

The surface morphology of the M-12 base membrane and PG-30 developed base membrane were studied using SEM and AFM. As shown in Figure 1, the surface morphology of the original base membrane and the expanded base membrane was observed and compared with the SEM images Figure 1a,c, Figure 1b,d of the original base membrane and expanded base membrane. It was found that the surface bulge of (c) was more obvious than that of Figure 1a, and the surface of Figure 1d had obvious spherical protrusions. AFM shows that the surface of the base membrane in Figure 2B is slightly more convex than that of the original membrane in Figure 2A. The average surface roughness values (Ra), which were quantified over 10 μm × 10 μm samples, were 92, 106 nm for the topography of the original PVDF/PVC (A) and PVDF/PVC basement membrane after PG-30 unfolding (B), respectively. The CA test results of the M-12 base membrane and PG-30 base membrane show that the contact angle of the PVDF-PVC original base membrane is about 90°. The contact angle of the base membrane after the expansion of PG-30 is about 105°.

The results of CA, SEM, and AFM showed that the surface of the M-12 base membrane changed after it was expanded by PG-30. It was speculated that after the base membrane was expanded at PG-30, DMF in the solvent would dissolve part of PVDF and PVC on the surface of the membrane, making the long chain of PVDF and PVC extend, making the surface roughness and hydrophobicity of the base membrane become larger. At the same time, the long chain on the surface of the polymer can be better cross-linked with the polymer in the coating layer after the expansion of the developing agent, so as to improve the firmness between the base membrane and the coating layer.

Polyethylene glycol with a low degree of polymerization can alleviate the solubility of PVDF and PVC, so the anti-dissolution agent is selected. The experimental results show that polyethylene glycol 200 (PG) has a good effect on the dissolution and expansion of the base membrane when the proportion of polyethylene glycol 200 (PG) to DMF and PG is 30% (PG-30). The expansion reagent of the PG-30 base membrane is selected under comprehensive consideration.

### 2.3. Performance Characterization of D-PP/PP Composite Membrane

**Figure 3:**
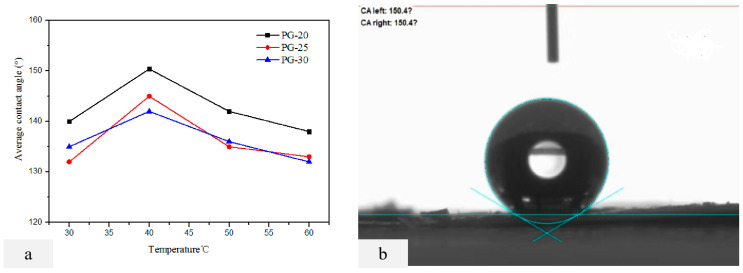
(**a**) The PVDF-PVC concentration of the coating layer is 4 wt%; PG-20, PG-25, and PG-30 were used as the casting agent, respectively. The contact angle images of the double layer membrane with 60 °C, 50 °C, 40 °C, and 30 °C scraping membrane in the casting liquid. (**b**)The PVDF-PVC concentration of the coating layer is 4 wt%, PG-20 is the casting membrane agent, and the contact angle image of the double layer membrane scraped at the casting liquid is 40 °C.

Four bottles of 4 wt% PVDF-PVC (85:15) coating materials were prepared with PG-20, PG-25, and PG-30 as the casting agent in the experiment. The casting solution was coated on the substrate at 60 °C, 50 °C, 40 °C, and 30 °C, respectively. PG-30 was used as the base membrane developing solvent, and a total of 12 double-layer composite membranes were prepared. A test of the CA of the double-layer membrane was carried out by the 3.3 methods to evaluate the hydrophobicity of the membrane. The results are shown in Figure 3a. When the PVDF-PVC concentration of PG-20, PG-25, and PG-30 casting solution is 4 wt%, the contact angle of the double-layer membrane changes greatly. PG-30 is the developing agent of the base membrane, PG-20 is the casting agent, and the concentration of the casting solution is 4 wt% PVDF-PVC coating. The contact angle of the double-layer membrane prepared by scraping the membrane at 40 °C of the casting solution is the largest, and its value is 150.4 ± 0.3°(Figure 3a). However, when the membrane preparation temperature is 50 °C and 60 °C, the contact angle decreases. It is speculated that the heat carried by 4 wt% of the casting solution is too large, which dissolves part of the base membrane during scraping, expands the membrane aperture, and reduces the hydrophobic properties of the membrane.

**Figure 4:**
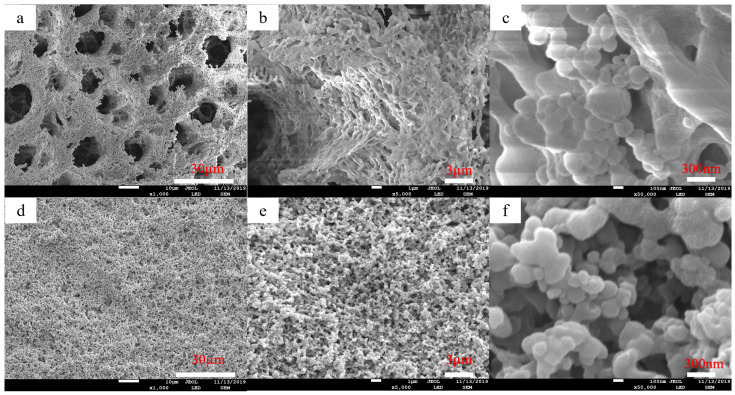
The SEM images of surface morphology of PVDF-PVC composite membranes with 4 wt% casting solution concentration ((**a**–**c**) magnified 500, 5000, 50,000, respectively). The PVDF-PVC concentration of the coating layer is 4 wt%, PG-20 as the casting agent, and the SEM image of the double layer of the scraping membrane at 40 °C of the casting liquid ((**d**–**f**) magnified 500, 5000, 50,000, respectively).

The coating layer is PVDF-PVC with a concentration of 4 wt% of the casting solution, and PG-20 is the casting agent. The double-layer membrane that was prepared by scraping the membrane at 40 °C of the casting solution has a contact angle of more than 150°, showing super-hydrophobicity. The surface morphology was observed by SEM. As shown in Figure 4f, it is found that the surface of the double-layer membrane prepared by the coating casting solution of 4 wt% PVDF-PVC is covered by cross-linked microspheres. The average diameter of the sphere is about 100 nm, and nanoscale microspheres are distributed on the microstructure surface of the double-layer membrane. Compared with the SEM images of M-4 prepared with the casting solution concentration of 4 wt% and the double layer membrane prepared with the coating solution concentration of 4 wt% PVDF-PVC (as shown in Figure 4c), the surface roughness of the double layer membrane is significantly higher than that of the M-4, but the contact angle is smaller than that of the M-4 (152°). It could be that there are too many pores on the surface of the double-layer membrane, and the water droplets exhibit the micro-capillary phenomenon on the membrane surface. The liquid and the membrane surface form a transition state between Wenzel and Cassie–Baxter wetting states.

It is reported that, as a semi-crystalline polymer, the precipitation of PVDF in the phase separation process is controlled by liquid–liquid phase separation and solid–liquid phase separation, accompanied by the crystalline cross-linked spherulite structure [5,29,30]. When the membrane casting solution that has a concentration of 4 wt% PVDF-PVC coating layer is immersed in the coagulation bath water; due to the low polymer concentration, the solvent DMF and non-solvent water at the coating layer/bath interface rapidly transfer mass, resulting in the non-solvent concentration in the membrane casting solution meeting the liquid–liquid phase separation requirements, so that the membrane casting solution of the coating layer has an instantaneous liquid–liquid phase separation. Although liquid–solid phase separation has advantages from the perspective of thermodynamics, the formation of a liquid nucleus in the casting solution with low polymer concentration is much faster than the formation of a crystal nucleus, and the liquid–liquid phase separation takes the dominant position when the liquid–liquid phase separation is carried out, some crystal nuclei grow slowly, forming nanoscale cross-linked spherical particles. Lin et al. [25]. At the same time, the addition of PG causes a large number of tiny aggregates to form in the coating solution, resulting in an increase in the contact angle of the composite membrane surface, forming a rough membrane surface, and improving the contact angle of the composite membrane. At the same time, both the coating solution and the membrane material of the base membrane are PVDF-PVC. Due to PG-20 being used as the casting agent, and a membrane casting solution with a PVDF-PVC concentration of 4 wt% being used as the surface coating, at 40 °C, a certain dissolution effect on the PVDF-PVC base membrane can be achieved (as shown in Table 2), which can better combine the surface coating and the base membrane, increasing the firmness of the surface coating and the base membrane.

### 2.4. Performance Characterization of D-PPG/PP Composite Membrane

#### 2.4.1. Characterization of Nano-Graphite Dispersion

**Figure 5:**
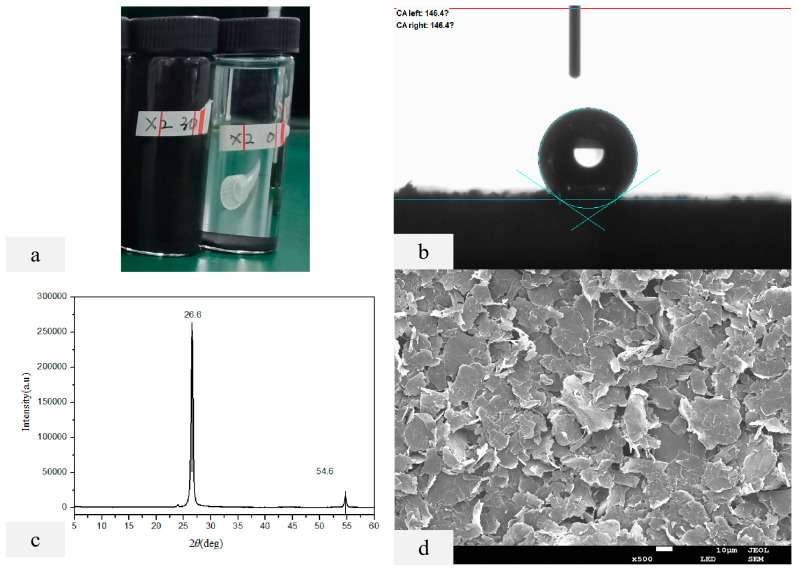
Nano-graphite dispersed by ultrasound after 10 days (**a**); CA of dispersed nano-graphite (**b**); XRD diffraction patterns of dispersed nano-graphite (**c**); SEM of dispersed nano-graphite (magnified 500) (**d**).

After 30 min of ultrasonic dispersion of nano-graphite and ten days of natural sedimentation, there was still no layering phenomenon, indicating that this method can obtain high concentration and stable nano-graphite dispersion. The contact angle of dispersed nano-graphite is around 146°, exhibiting good hydrophobicity. The XRD diffraction pattern of dispersed nano-graphite shows that the diffraction peaks of nano-graphite are at 26.6° (2θ) and 54.6° (2θ). The SEM results of dispersed nano-graphite also show that the dispersion effect of ultrasonic dispersed nano-graphite is good (Figure 5).

#### 2.4.2. Performance Characterization of Double Layer PVDF-PVC-Nano-Graphite/PVDF-PVC Composite Membrane

**Figure 6:**
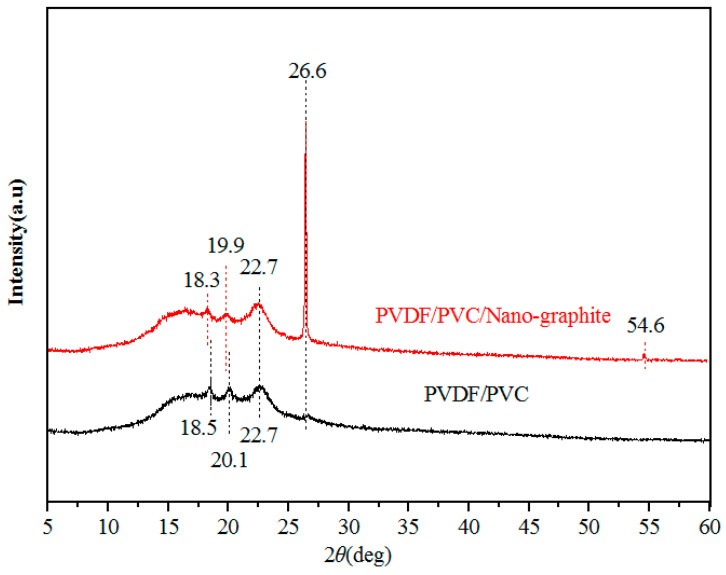
XRD of the PVDF-PVC membranes and PVDF-PVC/nano-graphite membranes.

The results of XRD show that the PVC is an amorphous compound and has no peak on the XRD curve, so the XRD diffraction pattern of the PVDF-PVC membrane shows only the peak of the crystalline polymer PVDF. Figure 6 shows the XRD diffraction patterns of the PVDF-PVC composite membrane and PVDF-PVC/nano-graphite composite membranes. For pristine PVDF-PVC composite membrane, the diffraction peaks near 2θ = 18.5°, 20.1°, 22.7°, and 26.6°, correspond to the γ-form of PVDF [30]. The addition of nano-graphite changes the diffraction peak of the composite membranes. For the PVDF-PVC/nano-graphite composite membranes, the diffraction peaks near 2θ = 18.3°, 19.9°, 22.7°, and 26.6°, are the characteristic peaks of the α-form of PVDF [31]. The incorporation of nano-graphite results in the transformation of PVDF crystals from gamma-shaped to alpha-shaped in the step-by-step process. Due to the characteristic diffraction peak of nano-graphite also being at 26.6°, the peak near 26.6° increases significantly. Previous studies reported that the incorporation of graphene nanosheets had impacts on PVDF crystallization. Due to the special affinity between PVDF and carbon surfaces, the graphene nanosheets worked as nucleating agents for PVDF precipitation and led to the PVDF crystalline transition [32,33], PVDF crystal transformation increases the roughness of the membrane surface.

**Figure 7:**
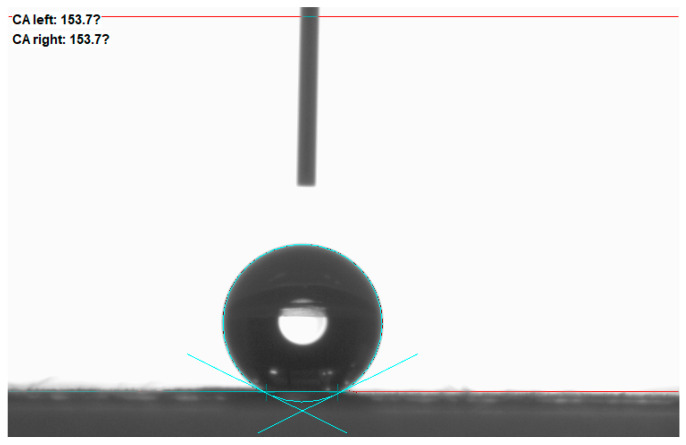
Contact angle of D-PPG/PP composite membrane.

As shown in Figure 7, the maximum contact angle of the nano-graphite-doped PVDF-PVC bilayer composite membrane reaches 153.7 ± 0.5°, and the rolling angle is less than 10°, indicating super-hydrophobicity. The contact angle results show that the contact angle of the PVDF-PVC double-layer membrane composite membrane doped with nano-graphite is higher than that of the PVDF-PVC double-layer membrane composite membrane (150.4 ± 0.3°). Research has found that doping nano-graphite in PVDF-PVC composite film can improve the hydrophobicity of the composite film. The doping of nano-graphite accelerates the polymer phase transition process in the coating layer, changes the PVDF crystal structure in the coating layer [33], and further improves the surface roughness of the film on the basis of the coating layer, thereby further improving the hydrophobicity of the composite membrane. The contact angle is increased from 150.4° to 153.7°, increasing by about 3°.

**Figure 8:**
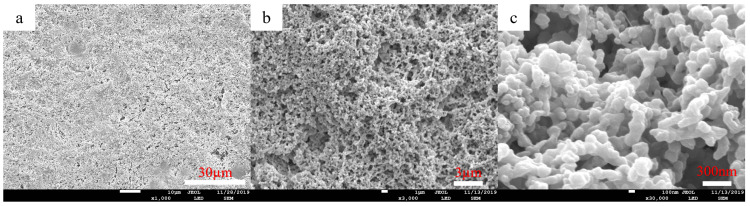
SEM images of D-PPG/PP composite membranes ((**a**–**c**) magnified 500, 5000, 50,000, respectively).

The surface morphology of nano-graphite doped PVDF-PVC bilayer composite membrane was studied through SEM. The SEM results are shown in Figure 8, and the surface of the nano-graphite doped PVDF-PVC bilayer composite membrane is covered by cross-linked microspheres. The diameter of the ball is about 30–300 nm, and it presents micro to nano-sized microspheres distributed on the surface of the bilayer membrane. Compared with the SEM of the PVDF-PVC bilayer composite membrane (Figure 4), the surface micro–nano level microsphere layer of the PVDF-PVC bilayer membrane doped with nano-graphite is more obvious, and the size of the nanospheres on the membrane surface is smaller. The doping of nano-graphite accelerates the polymer phase transition process in the coating layer, changes the PVDF crystal structure in the coating layer [34], and further improves the surface roughness of the membrane on the basis of the coating layer. The results are consistent with the contact angle.

### 2.5. Mechanical Properties of Composite Membranes

The mechanical properties of the PVDF-PVC original membrane, PVDF-PVC expansion membrane, the D-PP/PP composite membrane, and the D-PPG/PP composite membrane were tested. The results are shown in Table 3. The maximum tension of the double-layer composite membrane is better than that of the PVDF-PVC original membrane and expansion membrane. The maximum tensile force of the D-PP/PP composite membrane is 2.34 N, which is 19.4% higher than that of the PVDF-PVC base membrane (1.96 N). The maximum tensile force of the D-PPG/PP composite membrane is 2.39 N, which is 21.9% higher than that of the PVDF-PVC base membrane (1.96 N). The results of mechanical properties show that the mechanical strength of the double-layer composite membrane is improved to a certain extent compared with the original PVDF-PVC membrane [35]. The results of the contact angle and scanning electron microscope show that the double-layer composite membrane has the super-hydrophobic property of the coating. The mechanical properties of the D-PP/PP(D-PPG/PP) composite membrane can be improved to a certain extent by the preparation of a double-layer composite membrane, and the super-hydrophobic properties of the coating can be obtained at the same time. Therefore, it is feasible to prepare a D-PP/PP super-hydrophobic composite membrane with high mechanical strength by the coating-immersion phase separation method.

### 2.6. The Self-Cleaning Experiment of the D-PP/PP Super-Hydrophobic Composite Membrane

**Figure 9:**
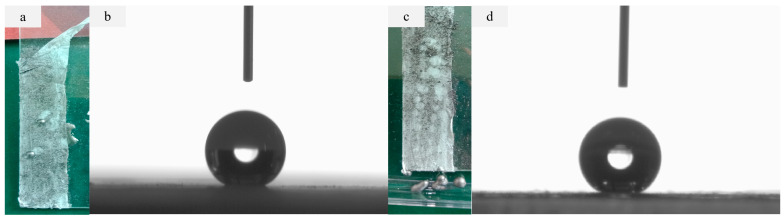
Self-cleaning test of the composite membrane surfaces: (**a**) demonstration of self-cleanability of the D-PP/PP membrane, which was dusted by randomly spraying graphite powder; (**b**) contact angle of the D-PP/PP membrane after the self-cleaning test (~150°); (**c**) demonstration of self-cleanability of the D-PPG/PP membrane which was dusted by randomly spraying graphite powder; (**d**) contact angle of the D-PPG/PP membrane after the self-cleaning test (~154°).

As far back as 2000 years ago, people found that some plants grow in mud, but their leaves are almost always clean. A typical example is the lotus leaf. The water droplets on the lotus leaf surface are almost spherical and can roll freely in all directions, while taking away the dust on the lotus leaf surface, showing a good self-cleaning effect. Scientists call this self-cleaning phenomenon the “lotus leaf effect” [36,37]. The super-hydrophobic polymer membrane is prepared by imitating the “lotus leaf effect” of the membrane; water can gather on its surface to form water droplets and flow down, and all kinds of polluting powder and water-based dye on the surface will be taken away by water so that the membrane surface can be naturally cleaned [38,39,40]. The SEM research results indicate that the surface of the double-layer composite membrane with a casting concentration of 4% is covered by cross-linked micro–nano-sized microspheres, and the microstructure is similar to that of lotus leaves. Among them, the average diameter of the surface microspheres on the D-PP/PP membrane is about 100 nm, and the average diameter of the surface microspheres on the D-PPG/PP membrane is about 50 nm. The result corresponds to the contact angle, the contact angle of the D-PP/PP membrane is 150.4 ± 0.3° and the D-PPG/PP membrane is 153.7 ± 0.5°. The self-cleaning experiment of a double-layer super-hydrophobic membrane shows that it has good self-cleaning performance. The self-cleaning test of double-layer composite membrane is shown in Figure 9. The self-cleaning performance of the D-PP/PP composite membrane was studied by spraying nano-graphite powder on the surface of the inclined membrane (inclined angle < 5°). Spray graphite powder randomly on the inclined membrane surface. When water droplets are added to the membrane surface, the water droplets will form a ball, and the graphite powder and water droplets will roll down at the same time [41,42], leaving a clean surface, as shown in Figure 9a. After three times of self-cleaning experiments, the static contact angle of the D-PP/PP membrane is about 150°, and it still shows good super-hydrophobicity, as shown in Figure 9b. As shown in Figure 9c, after three times of self-cleaning experiments, the static contact angle of the D-PP/PP membrane is about 153°, and it still shows good super-hydrophobicity, as shown in Figure 9d.

## 3. Materials and Methods

### 3.1. Materials

Polyvinylidene fluoride (PVDF, FR904) was purchased from Shanghai 3F New Materials Technology Co., Ltd.; Dimethylformamide (DMF, Analytical pure) was purchased from Aladdin Reagent Co., Ltd. (Shanghai, China); polyvinyl chloride (PVC) was purchased from Aladdin Reagent Co., Ltd.; polyethylene glycol 200 (PG, analytical pure) was purchased from National Pharmaceutical Group Chemical Reagent Co., Ltd. (Shanghai, China); nano-graphite (XFQ022) was purchased from Su Xianfeng Nanomaterials Technology Co., Ltd. (Nanjing, China); deionized water (self made).

### 3.2. Methods

#### 3.2.1. Preparation of the Basal Membrane

PVDF and PVC were vacuum-dried at 60 °C for 12 h before use. PVDF-PVC mixture was dissolved in DMF, then stirred at 60 °C for 6 h, and the membrane solution of PVDF-PVC at 12 wt% (85:15) (M-12) was prepared and degassed for more than 30 min. The composite membrane was prepared by NIPS, and about 10 mL of the cast membrane was scraped on MEMCAST^TM^ (Porometer Ltd., Nazareth, Belgium) with a knife gap of 200 µm. After scraping, the cast membrane was exposed to the air for 10 s and then immersed into a 25 °C cold pure water coagulation bath for 5 min. After the flat membrane was solidified, it was soaked by ultrasonic cleaning with anhydrous ethanol for 30 min, washed with deionized water, and then soaked in deionized water for 12 h. The residual solvent was removed and dried at air temperature. Finally, the membrane is dried in a vacuum oven of 60 °C for 6 h before use.

#### 3.2.2. Selection of Solvent Concentration

Through a literature search, it was found that polyethylene glycol with a low degree of polymerization can alleviate the solubility of PVDF and PVC in DMF. Therefore, polyethylene glycol 200 (PG) antidissolver [8,9] was selected. However, the concentration of PG has yet to be investigated. It is necessary to meet the requirements of dissolving the coating material, and at the same time, the PVDF-PVC base membrane should not be dissolved as much as possible.

##### Dissolution Test of Coating Material

PVDF and PVC were vacuum-dried at 60 °C for 12 h before use. The casting agent is DMF, the anti-solving agent is polyethylene glycol 200 (PG), and the casting solution concentration of the coating layer is 4 wt% PVDF-PVC (85/15). Seven groups of solutions with PG: DMF of 10:90, 15:85, 20:80, 25:75, 30:70, 35:65, and 50:50 (recorded as PG-10, PG-15, PG-20, PG-25, PG-30, PG-35, and PG-50, respectively) were prepared and placed in the bottle. Place the powder with a mass concentration of 4 wt% PVDF-PVC (85/15) in seven groups of solutions and place it in a water bath clock. Raise the temperature of the water bath kettle from 30 °C to 40 °C, 50 °C, and 60 °C. Observe the dissolution of the powder.

##### Dissolution Test of Base Membrane

In order to prevent the dissolution of the base membrane before scraping, DMF is used as the membrane casting agent, PG is the anti-solvent agent, and M-12 is used as the base membrane (the base membrane is cut to 15 mm × 15 mm for standby). Seven groups (bottles) of solutions (PG-10, PG-15, PG-20, PG-25, PG-30, PG-35, and PG-50) are prepared for the experiment, respectively. Put the cut base membrane into seven groups of solutions, put it into the water bath, and change the temperature of the water bath from 30 °C to 40 °C, 50 °C, and 60 °C. Observe the dissolution of the basement membrane within 30 min.

##### Base Membrane Expansion Test

In order to prevent the deformation of the base membrane in the solvent, and at the same time, it is hoped that the polymer PVDF and PVC on the surface of the base membrane will have a certain extension in the developing agent, so as to increase the adhesion between the two layers, the base membrane expansion experiment is designed. According to Section 3.2.2., the M-12 base membrane (60 × 60 mm) was soaked in different temperatures and solvent ratios for 3 min, then immersed in purified water, then immersed the membrane in ethanol solution for 1 h, and then ultrasonic at 60 °C for 1 h in the ultrasonic cleaner. Take out the membrane and wash the ethanol with purified water, dry it naturally, and dry it in a vacuum at 60 °C for 8 h. Measure the contact angle and surface morphology of the base membrane after expansion.

#### 3.2.3. Preparation of D-PP/PP Super-Hydrophobic Composite Membrane

##### Preparation of Upper Casting Solution

Dry PVDF and PVC in a vacuum at 60 °C for 12 h for standby. PVDF and PVC are used as membrane materials, and polyethylene glycol (PE200) and DMF in different proportions are used as solvents. The total mass of the membrane material is 4%. PVDF-PVC (*w*/*w* = 85/15) is dissolved in different proportions of polyethylene glycol (PE200) and DMF as the solvent, stirred at a constant speed for about 2 h at 60 °C, and then ultrasonically defoaming at 60 °C for more than 30 min to obtain the casting solution.

##### Preparation of Base Membrane

Immerse the M-12 base membrane in the optimum temperature and solvent ratio for 3 min, and then immerse it in pure water. Then, immerse the membrane in ethanol solution for 1 h, and then an ultrasound at 60 °C for 1 h in the ultrasonic cleaner. Take it out and wash the ethanol with pure water, dry it naturally, and dry it in a vacuum at 60 °C for 8 h.

##### Preparation of D-PP/PP Composite Membrane

**Figure 10:**
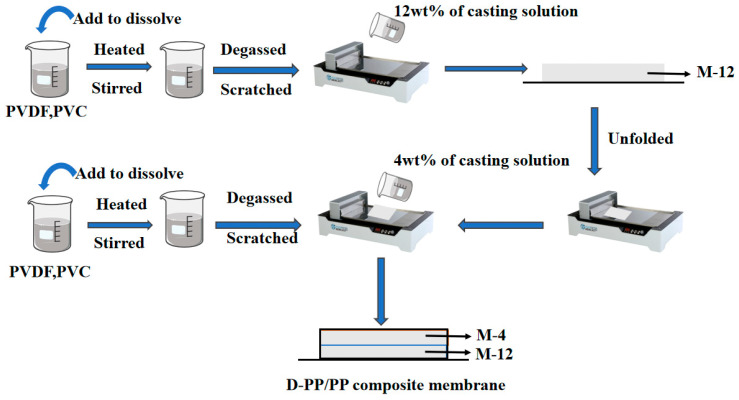
Preparation process diagram of D-PP/PP super-hydrophobic composite membrane.

As shown in Figure 10, the 4 wt% PVDF-PVC (*w*/*w* = 85:15) coating material membrane casting solution with the membrane casting agent was used, and the membrane casting solution was coated on the substrate at 60 °C, 50 °C, 40 °C, and 30 °C, respectively. After the completion of scraping, immerse the membrane in pure water. After the membrane is completely separated from the scraping template, immerse the membrane in ethanol solution for 30 min, and then ultrasound for 30 min at a constant temperature of 60 °C in the ultrasonic cleaner. Take it out, wash the ethanol with pure water, and then soak it in deionized water for 12 h, remove the residual solvent, and let it dry naturally. Dry it for 6 h under the temperature control of 60 °C in the vacuum drying box, and wait for standby.

#### 3.2.4. Preparation of the D-PPG/PP Super-Hydrophobic Composite Membrane

##### Preparation of Nano-Graphite Dispersion

In order to prevent the aggregation of nano-graphite in solution and further reduce the number and size of nano-graphite layers, a high-power ultrasound instrument was selected to disperse the nano-graphite through ultrasound. Add 2.5 g nano-graphite into 500 mL DMF solution (initial concentration of nano-graphite: 5 mg/mL), and ultrasonic peel it at 50 °C for 30 min.

##### Preparation of Upper Casting Solution

Dry PVDF and PVC, and wait for 12 h in a vacuum at 60 °C. A casting solution containing 1% nano-graphite and 4% PVDF-PVC (85%/15%) was prepared using PVDF, PVC, and nano-graphite as membrane materials, polyethylene glycol (PE200), DMF, and nano-graphite dispersion as solvents.

##### Preparation of Base Membrane

Preparation of base membrane according to the method outlined in Section 3.2.3.

##### Preparation of the D-PPG/PP Composite Membrane

**Figure 11:**
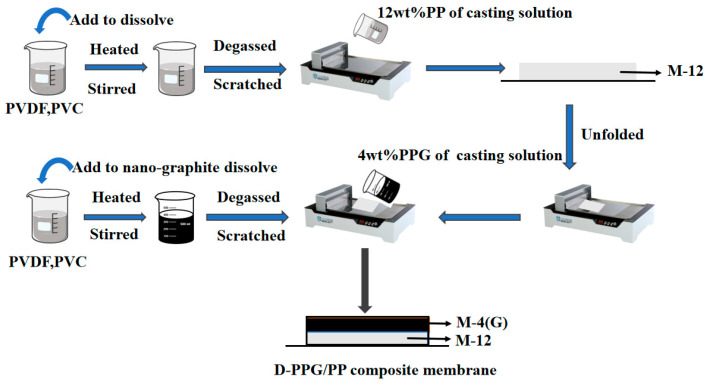
Preparation process diagram of D-PPG/PP super-hydrophobic composite membrane.

As shown in Figure 11, a casting solution containing 1% nano-graphite and 4% PVDF-PVC (85%/15%) with the membrane casting agent was used, and the membrane casting solution on the substrate was coated. After the completion of scraping, immerse the membrane in pure water. After the membrane is completely separated from the scraping template, immerse the membrane in ethanol solution for 30 min, and then ultrasound for 30 min at a constant temperature of 60 °C in the ultrasonic cleaner. Take it out, wash the ethanol with pure water, and then soak it in deionized water for 12 h, remove the residual solvent, and let it dry naturally. Dry it for 6 h under the temperature control of 60 °C in the vacuum drying box, and wait for standby.

### 3.3. Characterizations

The morphology of the PVDF-PVC membrane surface was investigated using Scanning Electron Microscopy (SEM) and, Scanning Probe Microscope (SPM). The surface morphology of the composite membranes was characterized by JSM-7100F field emission SEM (JEOL, Akishima, Japan). The surface roughness of samples was tested by using atomic mechanical microscopy (SPM). The wettability and hydrophobicity of the composite membrane can be known by measuring the contact angle (CA) of the composite membrane, using the CA goniometer (OCA15EC, SCA20 software, Stuttgar, Germany). The crystal structure of the sample was determined by Wide-angle X-ray Diffraction (WAXD) using an X-ray diffractometer (Ultima Ⅳ, Rigaku, Akishima, Japan).

## 4. Conclusions

In order to reduce the cost of the PVDF membrane and improve its hydrophobicity, PVC was added to PVDF by using the NIPS method, and the hydrophobicity of the composite membrane was improved by changing the concentration of PVDF-PVC (85:15) composite membrane casting solution. The contact angle of the PVDF-PVC composite membrane prepared at the concentration of 4 wt% of casting solution can reach 152°, and the rolling angle is less than 5°, which shows super-hydrophobicity. However, the mechanical properties of PVDF-PVC super-hydrophobic composite membrane prepared with 4 wt% casting solution were poor. The contact angle of PVDF-PVC composite membrane prepared at 12% casting solution concentration is only about 90°, but its mechanical properties are good [11]. In order to improve the mechanical properties of the super-hydrophobic membrane, the D-PP/PP super-hydrophobic composite membrane was prepared by the coating-immersion phase separation method, that is, on the PVDF-PVC base membrane with good mechanical properties and the casting solution concentration of 12 wt%, the PVDF-PVC coating layer with a casting solution concentration of 4 wt% was coated. The contact angle of the prepared D-PP/PP membrane was 150.4 ± 0.3° at most. The SEM results showed that the surface of the D-PP/PP membrane was covered by cross-linked microspheres with a diameter of about 100 nm; nano-scale microspheres are distributed on the microstructure surface of the double-layer membrane. The results of mechanical properties showed that the maximum tensile strength of the D-PP/PP composite membrane was 2.34 N, which was 19.4% higher than that of the PVDF-PVC base membrane (1.96 N). In order to further improve the hydrophobicity and mechanical properties of the bilayer membrane, nano-graphite was added to the coating layer in the experiment. The experimental results showed that the coating layer was doped with 1.0% nano-graphite casting solution with a concentration of 4 wt% PVDF-PVC, and the maximum contact angle of the prepared double layer PVDF-PVC-nano-graphite/PVDF-PVC composite membrane reached 153.7 ± 0.5°, the contact angle increased by 3.3°. The SEM comparison showed that compared to the D-PP/PP composite membrane, the surface of the D-PPG/PP composite had a more obvious micro–nano level microsphere layer, and the size of the nano microsphere on the membrane surface was smaller (Figure 4f and Figure 8c). The mechanical properties show that the nano-graphite-doped PVDF-PVC double-layer composite membrane can withstand a maximum tensile force of 2.39 N, which is 21.9% higher than the PVDF-PVC base membrane (1.96 N). The mechanical properties of the D-PP/PP composite membrane can be improved to a certain extent by the preparation of the D-PP/PP composite membrane, and the super-hydrophobic properties of the coating can be obtained at the same time. Therefore, it is feasible to prepare a D-PP/PP super-hydrophobic composite membrane with high mechanical strength by the coating-immersion phase separation method. At the same time, it was found that adding nano-graphite to the coating layer can better improve the hydrophobic, mechanical, and self-cleaning properties of the D-PP/PP composite membrane.

## Figures and Tables

**Table 1:** Dissolution of coating.

Temperature/°C	PG-10	PG-15	PG-20	PG-25	PG-30	PG-35	PG-50
30 °C	Not Dissolve	Not Dissolve	Not Dissolve	Not Dissolve	Not Dissolve	Not Dissolve	Not Dissolve
40 °C	Not Dissolve	Not Dissolve	Not Dissolve	Part Dissolve	Part Dissolve	Not Dissolve	Not Dissolve
50 °C	Dissolve	Dissolve	Dissolve	Dissolve	Dissolve	Part Dissolve	Not Dissolve
60 °C	Dissolve	Dissolve	Dissolve	Dissolve	Dissolve	Dissolve	Not Dissolve

**Table 2:** Dissolution of basement membrane.

Temperature/°C	PG-10	PG-15	PG-20	PG-25	PG-30	PG-35	PG-50
30 °C	Dissolve	Not Dissolve	Not Dissolve	Not Dissolve	Not Dissolve	Not Dissolve	Not Dissolve
40 °C	Dissolve	Dissolve	Part Dissolve	Not Dissolve	Not Dissolve	Not Dissolve	Not Dissolve
50 °C	Dissolve	Dissolve	Dissolve	Part Dissolve	Not Dissolve	Not Dissolve	Not Dissolve
60 °C	Dissolve	Dissolve	Dissolve	Dissolve	Dissolve	Not Dissolve	Not Dissolve

**Table 3:** Mechanical properties of the composite membrane.

Membrane	Membrane Thickness (mm)	Maximum Tension of the Membrane (N)	Tensile Stress at Break (MPa)	Elongation at Break (%)	LEPw (MPa)
M-4 Original membrane	0.040 ± 0.014	0.013 ± 0.006	0.330 ± 0.004	11.667 ± 2.582	0.22
M-12 Original membrane	0.068 ± 0.005	1.96 ± 0.008	2.957 ± 0.016	156.67 ± 5.162	1.97
M-12 expansion membrane	0.072 ± 0.004	2.16 ± 0.001	3.006 ± 0.002	148.67 ± 3.233	1.86
D-PP/PP composite membrane	0.108 ± 0.015	2.34 ± 0.017	3.160 ± 0.011	158.16 ± 2.675	2.05
D-PPG/PP composite membrane	0.139 ± 0.011	2.39 ± 0.18	3.220 ± 0.011	169.15 ± 4.41	2.12

## Data Availability

Data are contained within the article.
